# Screening for MicroRNA combination with engineered exosomes as a new tool against osteosarcoma in elderly patients

**DOI:** 10.3389/fbioe.2022.1052252

**Published:** 2022-12-05

**Authors:** Jiyu Han, Zitong Zhao, Yanhong Wang, Tao Yu, Daqian Wan

**Affiliations:** ^1^ Department of Orthopedics, Tongji Hospital, School of Medicine, Tongji University, Shanghai, China; ^2^ Key Laboratory of Spine and Spinal Cord Injury Repair and Regeneration, Ministry of Education, Shanghai, China; ^3^ Department of Orthopaedic, Ruijin Hospital, Shanghai Jiao Tong University School of Medicine, Shanghai, China

**Keywords:** osteosarcoma, miR-449a, CCNB1, engineered exosomes, bioinformatics analysis, wet-lab experiment

## Abstract

The most common primary malignant bone sarcoma is Osteogenic sarcoma (OS) which has a bimodal age distribution. Unfortunately, the treatment of OS was less effective for elderly patients than for younger ones. The study aimed to explore a new microRNA (miRNA) which can bind to combining engineered exosomes for treatment of older OS patients. Based on GSE65071 and miRNet 2.0, two up-regulated miRNAs (miR-328, miR-107) and seven down-regulated miRNAs (miR-133b, miR-206, miR-1-3p, miR-133a, miR-449a, miR-181daysay, miR-134) were selected. Next, we used FunRich software to predict the up-stream transcription factors (TFs) of differentially expressed miRNAs (DE-miRNAs). By comparing target genes predicted from DE-miRNAs with differentially expressed genes, we identified 12 down-regulated and 310 up-regulated mRNAs. For KEGG analysis, the most enriched KEGG pathway was Cell cycle, Spliceosome, and Protein digestion and absorption. By using protein-protein interactions network, topological analysis algorithm and GEPIA database, miR-449a /CCNB1 axis was identified. Experiments *in vitro* were conducted to confirm the results too. MiRNA-449a is down-regulated in osteosarcoma and suppresses cell proliferation by targeting CCNB1. Our findings not only reveal a novel mechanism of miR-449a /CCNB1 in OS but also had laid the groundwork for further investigation and analysis in the field of exosome engineering.

## Introduction

The most common primary malignant bone sarcoma (SARC) is Osteogenic sarcoma (OS) (also called osteosarcoma) (Li et al., 2021a). It is known that OS has a bimodal age distribution with a first peak during adolescence and a second peak in older adulthood (Han et al., 2020). It is characterized by early metastasis and primary tumors that originate from mesenchymal cells (Zhang et al., 2020). Meanwhile, poor survival rates persist for OS patients, particularly the oldest old or very old (≥80 years) (Yang et al., 2021). Current treatment ways are less in the elderly compared with the younger patients (Wang et al., 2019). Due to the difficulty of administering appropriate therapy to older OS patients, studies have reported a worse prognosis for them *versus* younger patients (Hayakawa et al., 2020). Thus, understanding its pathogenesis and developing new treatments remain major unmet needs.

MicroRNAs (miRNAs) are highly conserved, chemically stable, small noncoding RNA molecules which have approximately 20–24 nucleotides (Wang et al., 2021a). MiRNAs are naturally expressed in all organs and cells and play an important role in post-transcriptional regulation through base-pairing to complementary sequences within targeted mRNAs to induce their degradation or suppressing translation (Chipman and Pasquinelli, 2019). There has been much research showing that many miRNAs which may play a role in OS tumor now (Soghli et al., 2022). For example, Xu et al., found hsa-miR-30d-5p, hsa-miR-17–5p, hsa-miR-98–5p, hsa-miR-301a-3p, and hsa-miR-30e-5p were the central hubs of the miRNA-mRNA networks and was also strongly implicated in OS (Xu et al., 2021). It is apparent that miRNAs are both potential biomarkers and potential therapeutic targets for OS.

Molecular changes in disease occurrence and development can be detected through bioinformatics methods, and are effective methods to study disease pathogenesis (Qiang et al., 2021; Wang et al., 2022a; Wang et al., 2022b; Qiang et al., 2022). In the current study, we first investigated miRNA and mRNA expression associated with OS using Gene Expression Omnibus (GEO), miRNet database. Using integrated miRNA and mRNA expression analysis, it is possible to construct a miRNA-mRNA interactive network to identify hub genes and provide additional insight into the pathogenesis of OS. In the next step, several experiments including cell apoptosis analysis, western blot analysis, and luciferase reporter assay were performed to determine the effects of the miRNA on the proliferation of SaOS-2 cells and regulate downstream target gene.

## Materials and methods

### miRNA microarray and data analysis

From the NCBI GEO database (http://www.ncbi.nlm.nih.gov/geo/), we were able to locate Osteosarcoma plasma datasets. We selected GSE65071 dataset (Allen-Rhoades et al., 2015) obtained using the GPL19631 platform (Exiqon miRNome platform, human panels I + II, V3) and excluded 10 metastatic samples. There are 10 samples from primary OS and 15 samples from healthy control individuals in this dataset.

In order to determine patients’ differentially expressed miRNAs (DE-miRNAs) with OS, the online tool GEO2R (https://www.ncbi.nlm.nih.gov/geo/geo2r) was used. The DE-miRNAs with significant fold changes were identified based on the significance threshold of *p* < 0.05 and |logFC (fold change) |>1.5. To further assess DE-miRNAs in OS, the ggplot2 package (Gustavsson et al., 2022) (version 3.3.3) was used for visualization. We constructed the heat map to visualize the levels of DE-miRNAs expression in GSE65071 dataset with Euclidean distance using ComplexHeatmap package (Gu and Hübschmann, 2021).

### Screening of DE-miRNAs’ targeted genes

DE-miRNAs’ target genes were screened using the miRNet 2.0 (https://www.mirnet.ca/miRNet/home.xhtml) (Chang et al., 2020). We first compared the DE-miRNAs based on GSE65071 dataset and osteosarcoma-associated miRNAs based on miR2Disease, HMDD, PhenomiR. By analyzing the result of the intersection, up- or down-regulated hub miRNAs were screened. Next, we remain chose miRNet 2.0 as a prediction tool for potential miRNA downstream targets.

### Screening of potential transcription factors

FunRich software (http://www.funrich.org)was used to predict up-stream transcription factors (TFs) of DE-miRNAs (Fonseka et al., 2021). FunRich was used to identify the top 10 TFs associated with DE-miRNAs, which were examined separately for up-regulated and down-regulated miRNAs.

### Screening of differentially expressed genes (DEGs)

From the NCBI GEO database, we were able to locate Osteosarcoma plasma datasets. We selected GSE14359 (Fritsche-Guenther et al., 2010) and GSE16088 (Paoloni et al., 2009) dataset obtained using the GPL96 platform (Affymetrix Human Genome U133A Array). There are eight samples from primary OS and 24 samples from healthy control individuals in this dataset. Table 1 provides information about the dataset.

**TABLE 1:** Details for GEO Osteosarcoma data.

References	Sample	GEO	Platform	Normal	Tumor
Fritsche-Guenther et al. (2010)	12	GSE14359	GPL96	2	10
Paoloni et al.(20018)	24	GSE16088	GPL96	8	16

In order to determine patients’ DEGs with OS, the online web-based tool GEO2R was used. The DEGs with significant fold changes were identified based on the significance threshold of *p* < 0.05 and |logFC (fold change) |>2.0. To further assess DEGs in OS, the ggplot2 package (Gustavsson et al., 2022) (version 3.3.3) was used for visualization. We constructed the heat map to visualize the levels of DEGs expression in GSE14359 (Fritsche-Guenther et al., 2010)and GSE16088 (Paoloni et al., 2009)dataset with Euclidean distance using ComplexHeatmap package (Gu and Hübschmann, 2021).

### Screening of target genes

Comparing DE-miRNAs’ targeted genes and DEGs predicted by miRNet and GEO respectively, Venn diagrams were generated. According to inverse correlation between target and miRNA, up-regulated and down-regulated target mRNA were defined.

### Functional annotation and pathway enrichment analysis

There are three components to Gene Ontology (GO) analysis: biological process (BP), cellular component (CC), and molecular function (MF). By using clusterProfiler R package (Yu et al., 2012) we analyzed the GO enrichment of DEGs.The Kyoto Encyclopedia of Genes and Genomes (KEGG) analysis was performed using the same R package as the GO pathway enrichment analysis. Aftwewards, we used the org. Hs.eg.db (version 3.4.0) and GO plot R (Walter et al., 2015) (version 1.0.2) packages for analysis and visualization of the results by generating cluster plots.

### Construction of PPI (protein-protein interaction) network and hub mRNAs

STRING (https://string-db.org/), one of the best-known online tools for predicting PPI, contains direct (physical) and indirect (functional) associations. The PPI network consists of protein complexes formed by biochemical events and/or electrostatic forces that serve a distinct biological function as a complex. The PPI network of an organism serves as a skeleton for its signaling circuitry, which mediates cellular response to environmental and genetic cues. Understanding this circuitry could improve the prediction of gene function and cellular behavior in response to diverse signals. With the help of the version 11.0 of PPI database STRING, we identified the DEGs involved in the PPI. The required interaction score for determining a significant interplay was medium confidence (0.400) as cut-off criteria in this network. As a second step, the PPI network was visualized with Cytoscape (version 3.8.2) (Doncheva et al., 2019). As a final step, the plug-in cytohubba was used to determine which DEGs were hub genes. The CytoHubba is a plug-in for the Cytoscape program, whose main functions was to screen out genes with carcinogenesis risk of the PPI network by using the MCC, MNC, Degree, EPC, Closeness, Radiality, Betweenness, and Stress.

### Identification of key gene

Further analyses were conducted using the intersection set of the eight methods. At present, there is no sufficient gene database for the detection of osteosarcoma. Because OS accounting for a large proportion of SARC, the GEPIA database (http://gepia.cancer-pku.cn/) of SARC is used. Based on the GEPIA database, the expression levels of the key gene and the survival result were further validated between SARC and normal samples.

### Cell line and culture

The American Type Culture Collection (ATCC) (Manassas, United States) provided the Human osteosarcoma SaOS-2 cells (ATCC^®^ HTB-85™) and the fetal human normal osteoblast cell line hFOB 1.19 (ATCC^®^ CRL-11372™) for use. 10% FBS (cat. No. 16140071), DMEM (cat. No. 30030) and McCoys 5A (modified) medium (cat. No. 16600108) were purchased from Invitrogen/Thermo Fisher Scientific, Inc. In addition to 10% FBS and antibiotics, McCoys 5A was used to culture the Saos-2 cells. We also cultured hFOB1.19 in DMEM with 10% FBS, 2.5 mM l-glutamine, and 0.3 mg/ml G418. Total cells were maintained at 37°C with 5% CO2 in a constant temperature incubator.

### Transfections

To increase or decrease miRNA expression, we obtained miR-449a mimic, miR-449a antisense oligonucleotide (ASO) and the respective scrambled negative controls (NCs) from GenePharma Co., Ltd. in Shanghai. The sequences of the miR-449a mimics and miR-449a ASOs were as follows: miR-449a mimics, 5′-UGG​CAG​UGU​AUU​GUU​AGC​UGG​U-3’; miR-449a ASOs, 5′-CCA​CGA​UGC​UAC​GUU​U-3’; miR-449a NC mimics, 5′-UUA​UCU​CCU​GUG​CGA​TT-3’; and miR-449a NC ASOs, 5′-CAG​UAC​AUU​GGU​UCU​GCA​A-3’. In 24-well plates at a density of 5 × 10^4^ cells/well, Lipofectamine 2000 (Life Technologies,cat. No. 11668019) was used for transfection. ASOs and mimics of miR-449a (final concentration, 300 nM) were diluted separately in 50 µl Opti-MEM™ Reduced Serum Medium (Gibco™ Thermo Fisher Scientific Inc. Waltham, MA, United States) without serum. Specifically, 1 µl Lipofectamine 2000 was added to 50 µl Opti-MEM Reduced Serum Medium, mixed gently, and incubated for 5 min at room temperature. In 20 min before transfection, miR-449a mimics and miR-449a ASOs were mixed with Lipofectamine 2000 and held at room temperature. The wells containing cells and medium were then added with miR-449a mimic- or ASO- Lipofectamine 2000 mixtures. Finally, transfected cells were incubated at 37°C for 20 h. By transfecting pcDNA3.1-CCNB1 plasmid into cells, CCNB1 overexpression was achieved. Shanghai GenePharma Co., Ltd. Synthesized and tested pcDNA3.1- CCNB1 plasmid and NC plasmid (containing a scrambled shNC sequence). We transfected plasmids (500 ng) into cells with lipofectamine 2000 as mentioned above: 37°C for 20  h, and then all cells were incubated at 37°C overnight.

### Quantitative reverse-transcription polymerase chain Reaction (qRT-PCR)

According to manufacturer’s instructions, total RNA was extracted from cell lines using TRIzol™ reagent (cat. No. 12183555) and reverse transcription was completed using the PrimeScript™ RT Reagent kit (Takara, Shiga, Japan). In accordance with manufacturer’s instructions, reverse transcription of total RNA into cDNA was performed with All-in-One™ miRNA First-Strand cDNA Synthesis kit (cat. No. 18091050). The expression of miR-449a and CCNB1 were assayed by using the SuperScript™ III Platinum™ SYBR™ One-Step Green qPCR kit (cat. No. 11736051) and TaqMan™ Fast Advanced Master Mix (cat. No. 4444557), respectively. The expression levels of miR-449a, and CCNB1 had been measured using the Applied Biosystems^®^ 7500 Fast Real-Time PCR System and accompanying Applied Biosystems^®^ 7500 Software (version 2.0.6). MiR-449a was internally referenced to U6, whereas CCNB1 was internally referenced to GAPDH. We used the following thermocycling conditions: Initial denaturation at 95°C for 10 min, followed by 45 cycles of denaturation at 95°C for 10 s, annealing at 60 °C for 30 s, and a final cycle of denaturation at 95°C for 10 s, annealing at 65°C for 60 s and extension at 97 °C for 1 s. The sequences of the primers used was GAPDH forward, GCC​TTT​GAT​GAC​TCA​GCT​CC and reverse, TTC​CTG​AAA​AGT​CAC​CAC​CC. In order to amplify U6, we used forward primer 5′-AGC​CCG​CAC​TCA​GAA​CAT​C-3′ (forward primer) and reverse primer 5′-GCC​ACC​AAG​ACA​ATC​ATC​C-3’ (reverse primer). Mir-449a was amplified using forward primers for TGC​GGT​GGC​AGT​GTA​TTG​TTA​GC and reverse primers CCA​GTG​CAG​GGT​CCG​AGG​T. CCA​AAT​CAG​ACA​GAT​GGA​AAT (forward primer) and GCC​AAA​GTA​TGT​TGC​TCG​A (reverse primer) were used for amplification of CCNB1. All unlabeled reagents and instruments were from Thermo Fisher Scientific, Inc.

### Target gene prediction and mutated site

Based on TargetScan version 7.2, potential binding sites for miR-449a and CCNB1 were identified. Based on conserved 8er and 6mer sites, TargetScan predicts miRNAs’ biological targets. Mutation of the 3′-untranslated region (3′-Umer, 7 mTR) of CCNB1 is generated by GeneArt™ (cat. No. A13282).

### Cell apoptosis analysis

The Dead cell apoptosis was determined by using the Annexin V-FITC/PI dead cell apoptosis kit (cat. No. V13242), following the Thermo Fisher Scientific, Inc’s instructions. In a nutshell, cells (1 × 10^6^ cells/tube) were harvested and washed once with phosphate buffered saline (PBS) with ice-cold. The cells next were centrifuged at 4°C for 5 min at 300× g. Then the supernatant liquid was removed and discarded after centrifugation. As a next step, cells were re-suspended in binding buffer 1X (100 µl) followed by the addition of annexin V-FITC (5 µl) and PI (1 µl). The BD FACSVerse™ flow cytometer and FACSuite™ software (BD Biosciences) was used to detect the apoptosis rate after a 15-min reaction at room temperature. Early apoptotic cells are found in the lower right quadrant, while cells in the upper right quadrant are late apoptotic cells. The apoptotic cell rate was equal to the sum of the two.

### Methyl thiazolyl tetrazolium (MTT) assay

We assessed cell proliferation by using MTT assay. SaOS-2 cells were seeded (5×10^5^ cells/well) into 96-well plates. Following this, 0.1 mg/ml of MTT reagent was added to the medium. In the next step, 100 µl dimethyl sulfoxide (DMSO) was used to dissolve the purple formazan crystals for 10 min. The optical density (O.D.) was determined at a wavelength of 570 nm by microplate reader (Multiskan Sky; Thermo Fisher Scientific, Inc.).

### Western blot analysis

The SaOS-2 cells were collected and lysed with radioimmunoprecipitation assay (RIPA) buffer (cat. No. 89900) containing protease inhibitor (cat. No. 78420). 0.2 ml cold RIPA lysis buffer was used to lyse 1×10^6^ cells *in vitro*. The mixture next was centrifuged at 4°C for 5 min at 300× g after it was stirred for 30 min at 4°C. In order to measure protein concentration, supernatant was collected and the BCA Protein Assay kit (cat. No. ab102536) was used. The protein (30 µg/lane) was separated using 12% SDS-PAGE gels for 2 h at 80 V. After protein transfer to polyvinylidene difluoride membranes, the proteins were blocked in 5% nonfat milk for 1 h at room temperature. The blocked membranes were next exposed to the primary antibodies for an overnight incubation at 4°C. At room temperature, membranes were incubated in HRP-conjugated secondary antibodies for 1 h after washing three times with 0.1% Tween 20 in PBS for 10 min each. Pierce™ enhanced chemiluminescence (ECL) western blot substrate (cat. No. 32209) was used to visualize protein bands. FluorChem System and AlphaView software were used for image acquisition and analysis.

### Luciferase reporter assay

Using a Dual Luciferase Reporter Assay System (Promega Corporation, cat. No. E1910), Luciferase activities were performed and analyzed. We amplified the 3′UTR of CCNB1 gene by PCR, and then cloned the luciferase gene downstream in the pGL/Promoter vector (Sangon Biotech Co., Ltd.) into the wild-type plasmids. To establish a control reporter for normalization, a Renilla luciferase reporter gene was used. The cells were then co-transfected with the CCNB1 3′UTR or mutant reporter construct containing luciferase, and miR-449a mimics by using Lipofectamine^®^ 2000 (cat. No. 11668019). Luciferase activity was assessed 24 h after transfection according to manufacturer’s protocol.

### Statistical analysis

Each experiment was repeated 3 times independently to ensure consistency. All statistical analyses were conducted by R statistical software (R Core Team, version 3.6.3) and GraphPad Prism (9.0) (GraphPad Software Inc.). All results were expressed as mean ± standard deviations (mean ± SD). The unpaired student’s t test was used to analyze differences between two groups. One-way ANOVAs are used to analyze differences among three groups, followed by Newman-Keuls post-hoc tests. A *p* value of less than 0.05 was considered to be statistically significant.

## Results

### Identification DE-miRNAs

**FIGURE 1:**
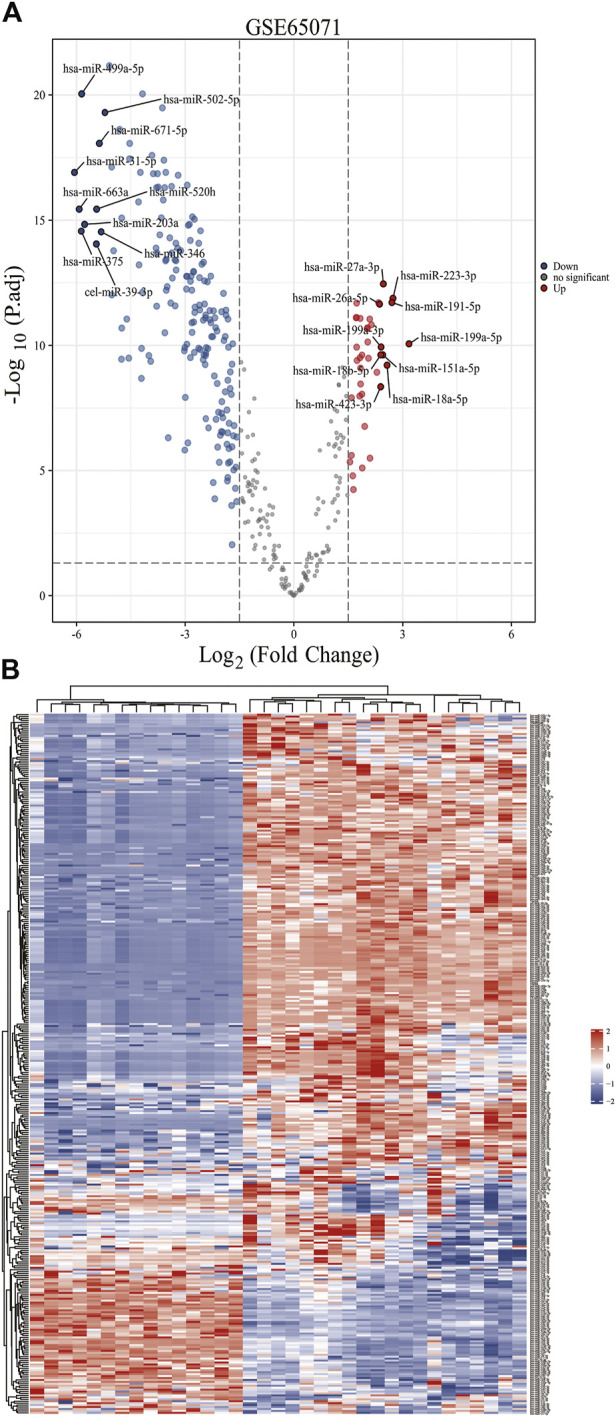
Identification of DE-miRNAs in OS and control samples. **(A)** The volcano plots and Heat maps showing all the expressed genes from GSE65071. **(B)** The Heat maps showing all the expressed genes from GSE65071. Blue represents downregulated genes, and red represents upregulated genes. Each column represents a probe, and each row represents a gene. DE-miRNAs: differentially expressed MicroRNAs.

Based on the analytical threshold, 212 DE-miRNAs between OS and control samples were screened out, including 38 up-regulated and 174 down-regulated miRNAs. The top 10 significantly up-/down- DE-miRNAs were shown in volcano plot (Figure 1A). All DE-miRNAs were shown in heatmap plot (Figure 1B).

### Identification target genes of DE-miRNAs

**FIGURE 2:**
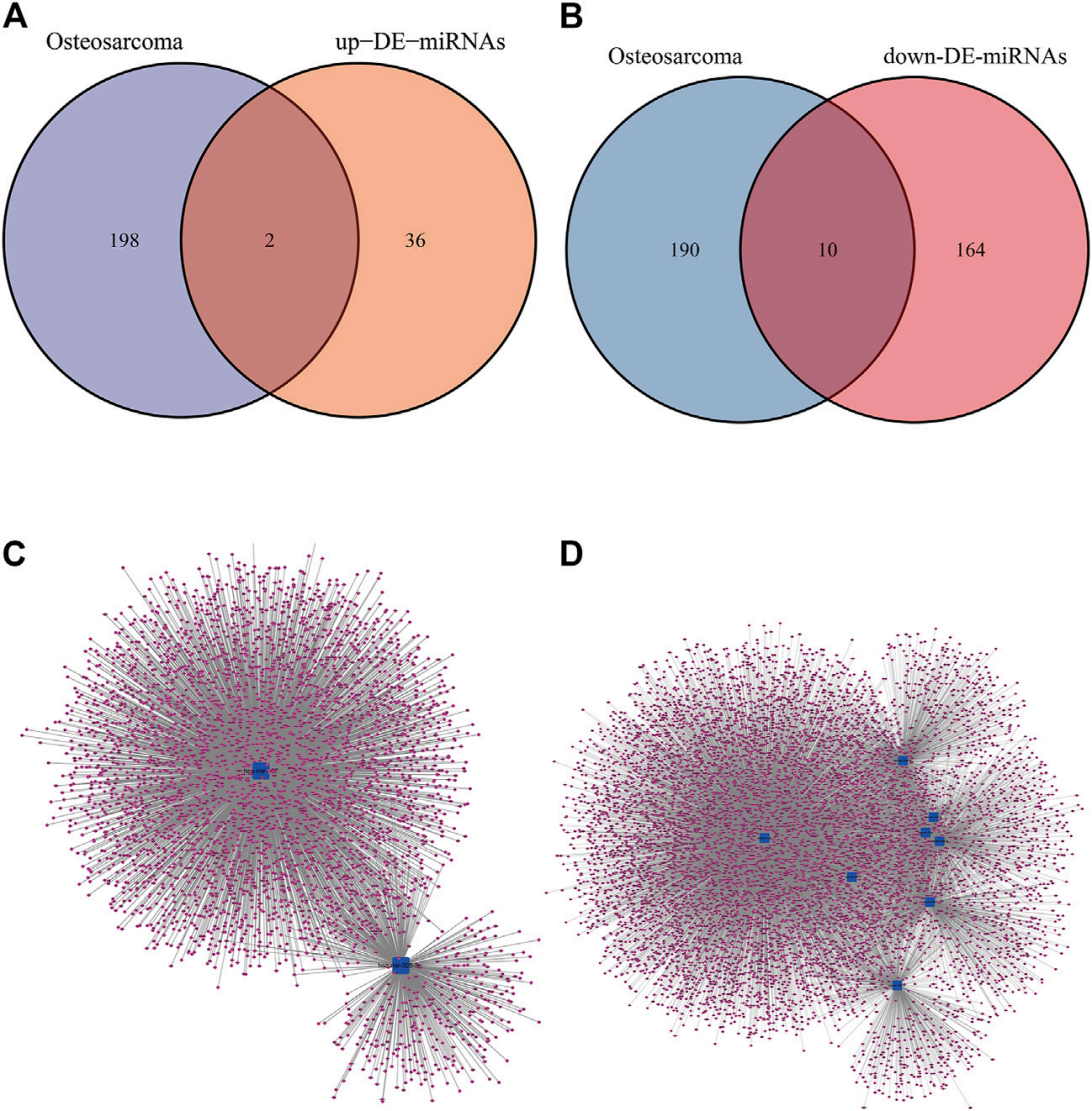
DE-miRNAs predicted potential target mRNAs in miRNet database. **(A)** The Venn diagram for the overlapping up-DE-miRNAs. **(B)** The Venn diagram for the overlapping down-DE-miRNAs. **(C)** The miRNA–mRNA networks of all up-regulated mRNAs. **(D) **The miRNA–mRNA networks of all down-regulated mRNAs.

Based on the results of comparison in Figures 2,2 up-regulated miRNAs (hsa-mir-328, hsa-mir-107) and 10 down-regulated miRNAs (hsa-mir-133b, hsa-mir-206, hsa-mir-329, hsa-mir-375, hsa-mir-1, hsa-mir-133a, hsa-mir-449a, hsa-mir-181daysay, hsa-mir-429, hsa-mir-134) were selected. Two up-regulated and ten down-regulated DE-miRNAs had successful target mRNA prediction, and 8493 up- and 3975 down-regulated mRNAs were identified. Cytoscape was used to construct a miRNA-gene network map (Figures 2, to better understand miRNA-target gene interactions in the osteosarcoma.

### Potential upstream TFs of DE-miRNAs

**FIGURE 3:**
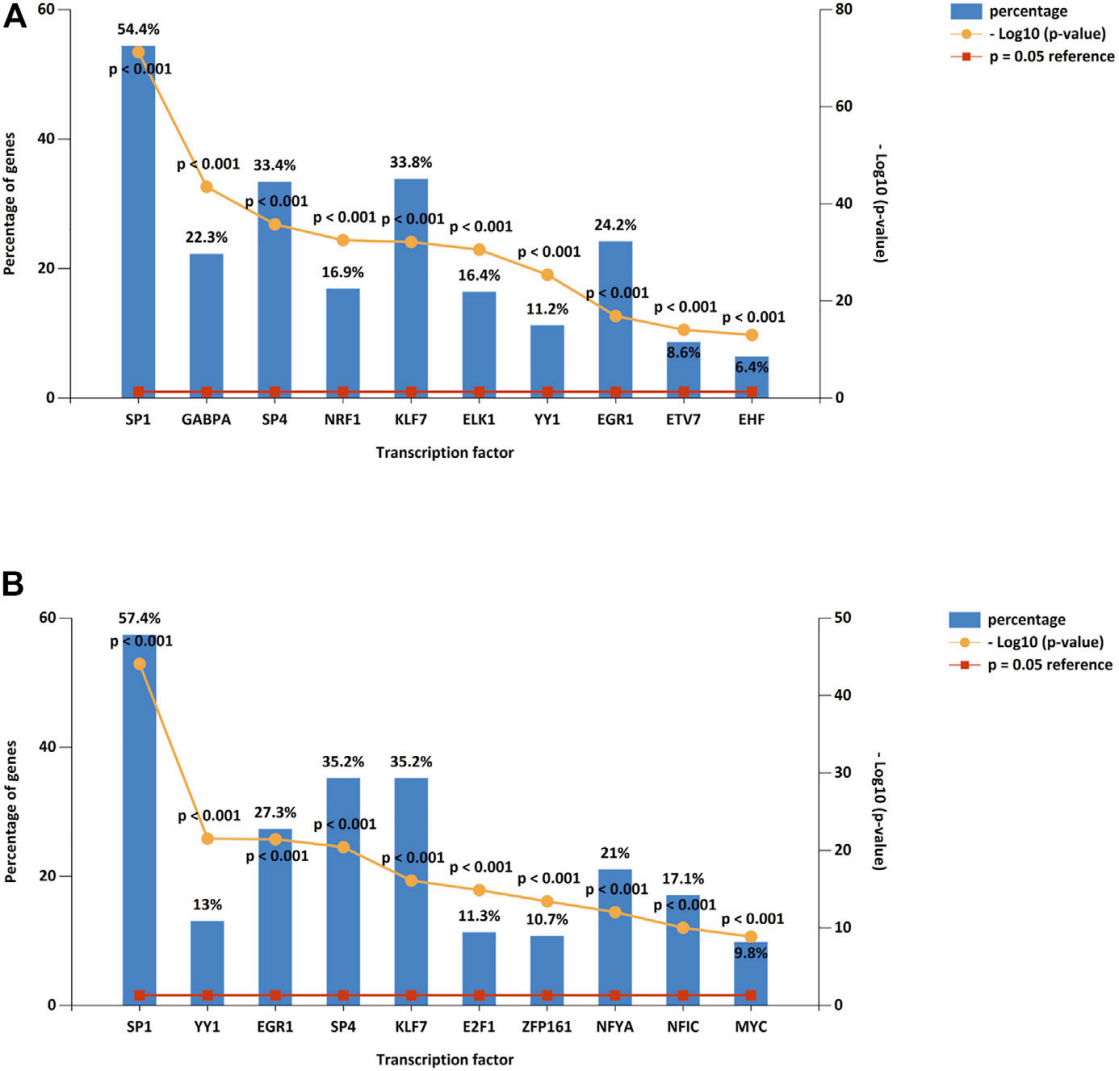
DE-miRNA upstreamTFs. **(A)** Top 10 TFs of down-regulated DE-miRNAs. **(B)** Top 10 TFs of up-regulated DE-miRNAs. TF: transcription factor.

We predicted upstream TFs of DE-miRNAs using FunRich software, and the first 10 predictions of up-regulated and down-regulated DE-miRNAs are shown in Figure 3 A and B. The top 10 TF of down-regulated DE-miRNAs were SP1, GABPA, SP4, NRF1, KLF7, ELK1, YY1, EGR1, ETV7, and EHF. There were ten transcription factors associated with up-regulated DEMs: SP1, YY1, EGR1, SP4, KLF7, E2F1, ZFP161, NFYA, NFIC, and MYC.

### Identification DEGs

**FIGURE 4:**
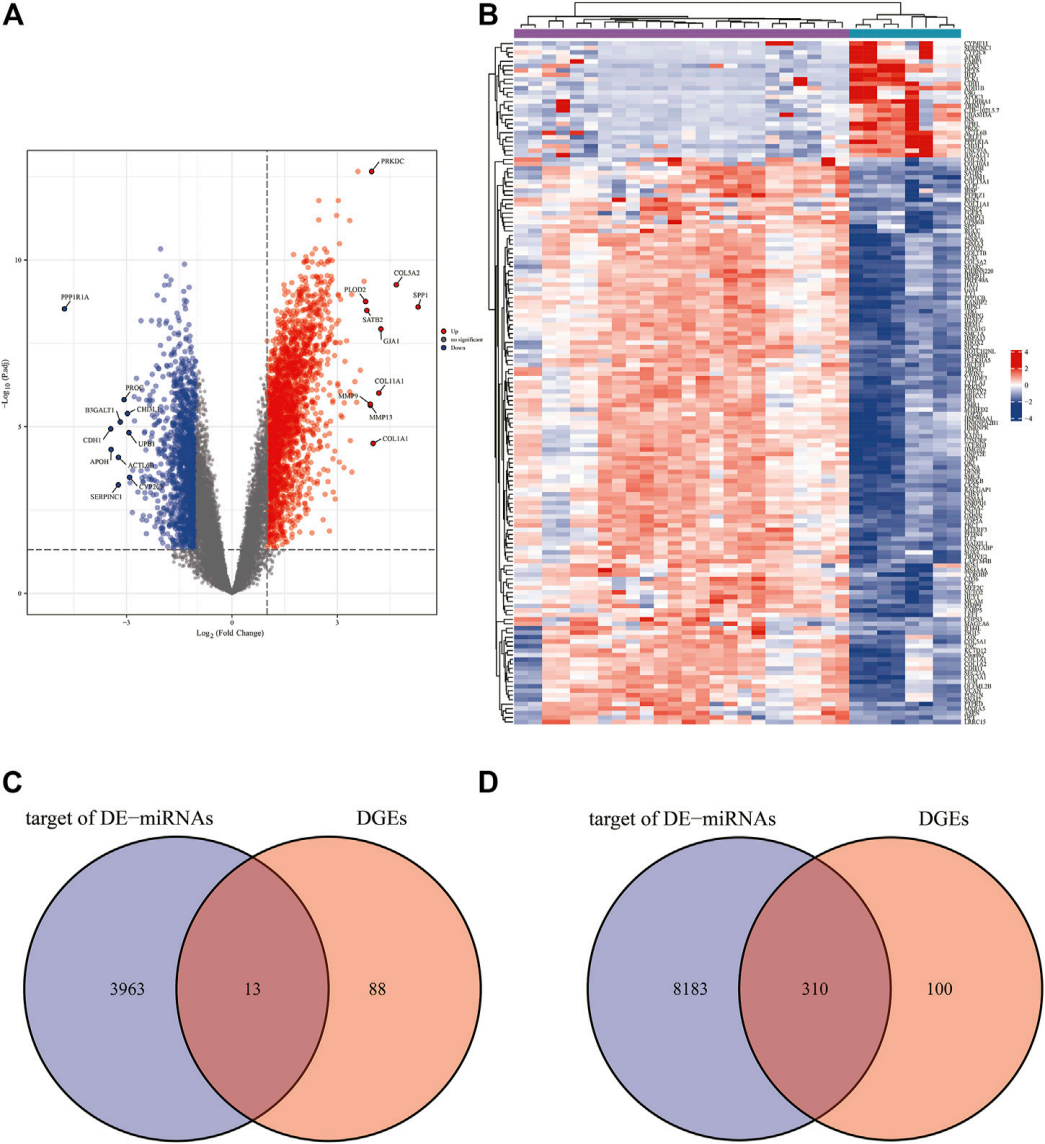
Predication of target genes. **(A)** The volcano plots and Heat maps showing all the expressed genes from GSE14359 and GSE16088. **(B)** The Heat maps showing all the expressed genes from GSE14359 and GSE16088. **(C)** The Venn diagram of mRNAs from the overlap between 101 down-regulated DEGs and 3975 target genes predicted from two up-regulated DE-miRNAs. **(D)** The Venn diagram of mRNAs from the overlap between 410 down-regulated DEGs and 8493 target genes predicted from two up-regulated DE-miRNAs.

Based on the analytical threshold, 511 DEGs between OS and control samples were screened out, including 410 up-regulated and 101 down-regulated miRNAs. The top 10 significantly up-/down- DE-miRNAs were shown in volcano plot (Figure 4A). All DE-miRNAs were shown in heatmap plot (Figure 4B).

### Identifying mRNAs

We compared 3975 target genes predicted from 2 DE-miRNAs that were up-regulated with 101 DEGs that were down-regulated, and the overlap 12 mRNAs were shown as a Venn diagram (Figure 4C). Venn diagram in Figure 4D shows that total numbers of 8493 target genes predicted from 10 down-regulated DE-miRNAs were compared with 410 DEGs that were up-regulated. In the overlapping region, 310 genes have been identified.

### GO functional annotation and KEGG pathway enrichment analysis

**FIGURE 5:**
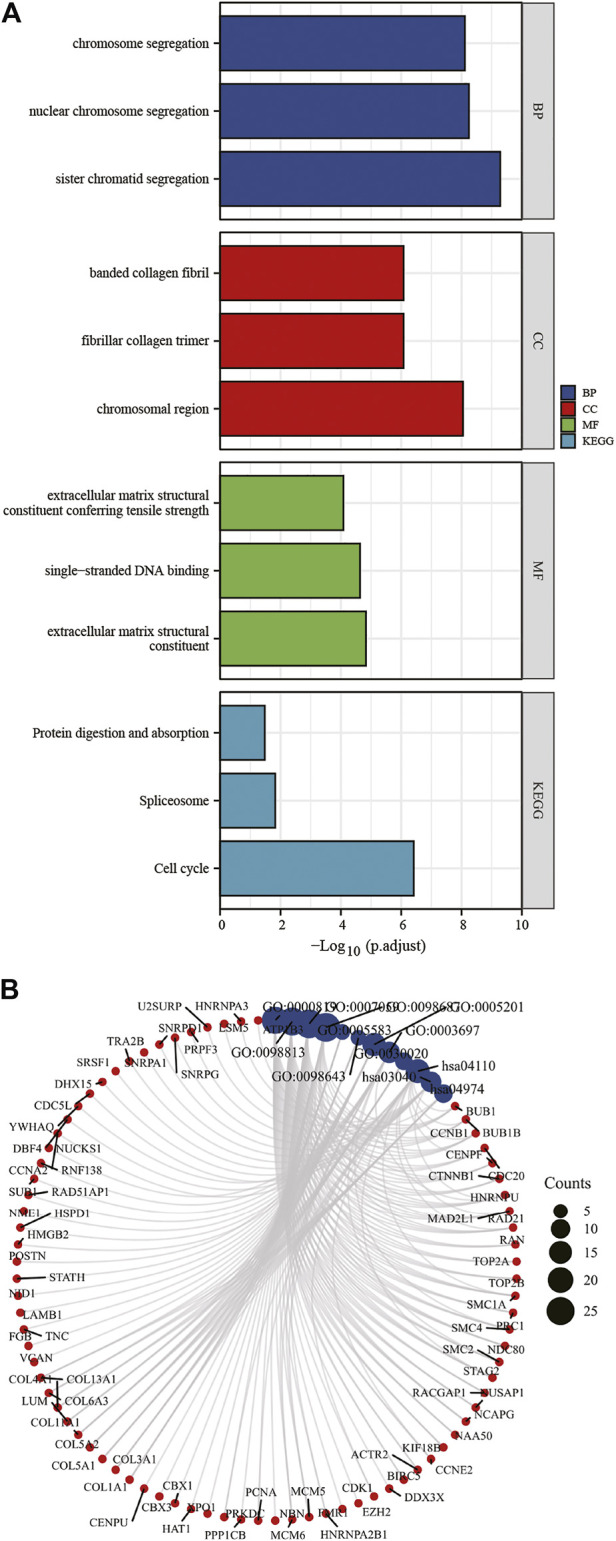
KEGG and GO enrichment analysis of DEGs **(A,B)**. DEGs, differentially expressed genes; GO, Gene Ontology; BP, biological process; CC, cellular component; MF, molecular function. KEGG, Kyoto Encyclopedia of Genes and Genomes.

To understand dysregulated miRNAs and mRNAs ' biological and functional meanings, GO and KEGG analysis was conducted (Figure 5A, Figure 5B). The GO functional annotation found that 322 DEGs overlapping with each other were enriched for 575 BP terms, 116 CC terms, and 58 MF terms. BP terms were significantly enriched in sister chromatid segregation, nuclear chromosome segregation, and chromosome segregation. For CC terms, DEGs were primarily enriched in chromosomal region, fibrillar collagen trimer, and banded collagen fibril. MF terms of these genes were mostly enriched in extracellular matrix structural constituent, single-stranded DNA binding, and extracellular matrix structural constituent conferring tensile strength. DEGs are significantly enriched in nine KEGG pathways, and most of them were significantly enriched in Cell cycle, Spliceosome, and Protein digestion and absorption.

### Construction of PPI network to distinguish critical hub genes

**FIGURE 6:**
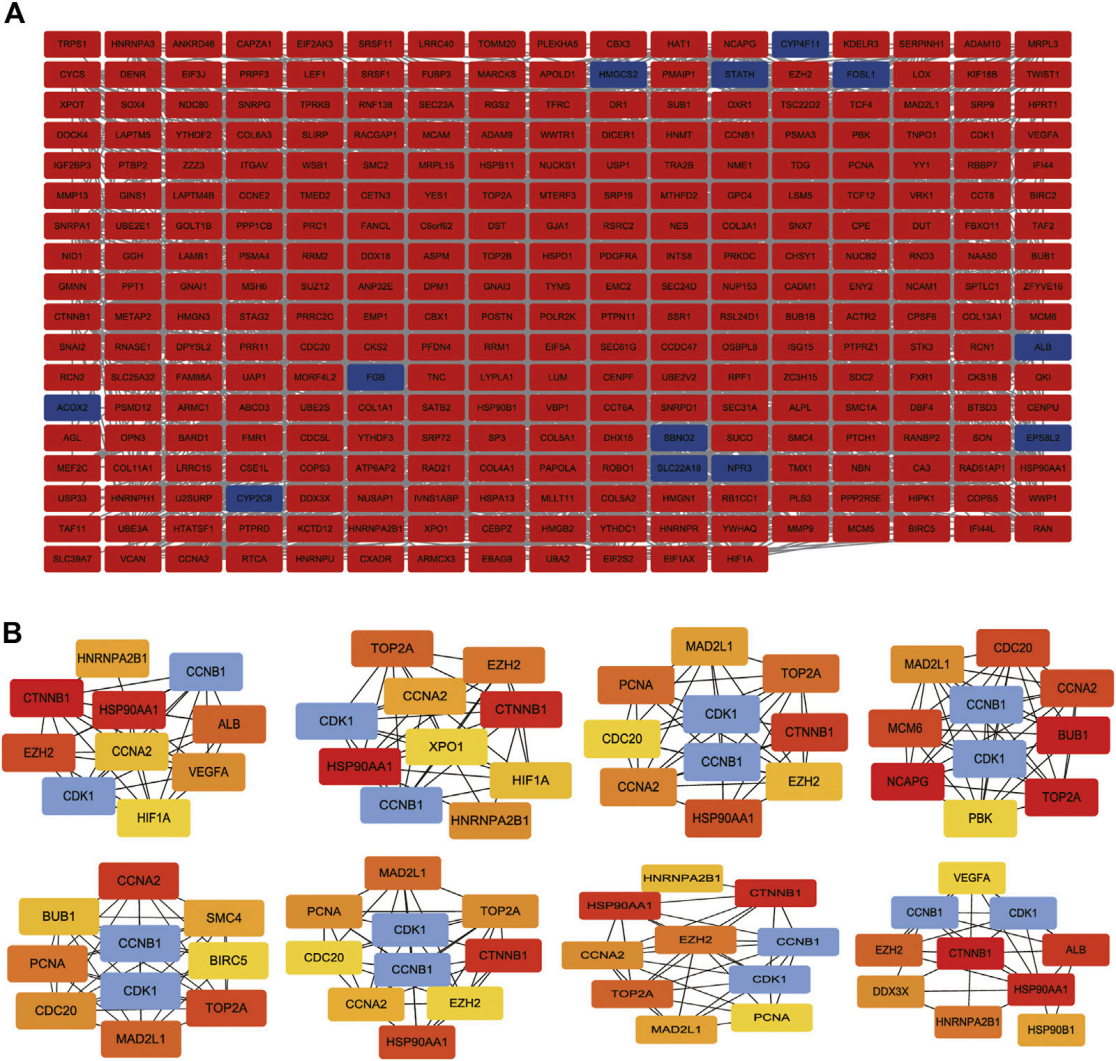
PPI network construction and analysis of hub genes. **(A)** The most significant module was obtained from the PPI network with 301 nodes and 2369 edges. **(B)** The hub genes were selected from the PPI network using the cytoHubba plugin. DEGs, differentially expressed genes; PPI, protein–protein interaction.

To construct and visualize PPI networks derived from candidate 322 DEGs, we used String database and Cytoscape software. We constructed the PPI network to explore the interactions among DEGs correlated with OS, consisting of 301 nodes and 2369 edges (Figure 6A). Moreover, the cytoHubba plugin selected 10 hub genes based on the PPI network. According to several topological analysis algorithms, nodes with a higher degree were selected for further analysis (Figure 6B).

### Identification of key mRNA and miRNA by GEPIA database

**FIGURE 7:**
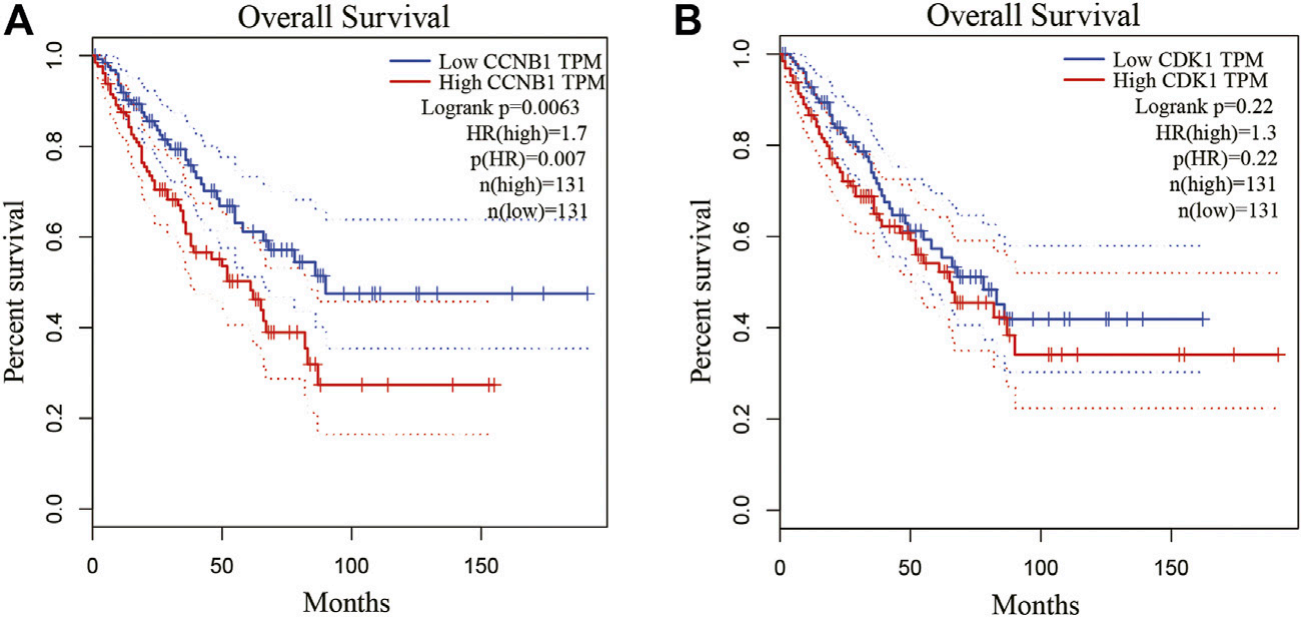
Kaplan-Meier survival curves of hub genes. **(A)** Kaplan-Meier survival curves of CCNB1 and CDK1. **(B)** The survival prognosis of the patients in the high-expression group was significantly worse than that of the patients in the low-expression group (*p* < 0.05).

Because Cyclin B1 (CCNB1) and Cyclin Dependent Kinase 1 (CDK1) had a large degree score and high repeat, they were chosen for further study by GEPIA database. CCNB1 and CDK1 have been next assessed with regard to survival in the GEPIA database. Based on our K-M curves (Figures 7, we found that high expression of CCNB1 predicted worse survival result in OS patients. In conclusion, CCNB1 had been identified as key genes in OS. According to the result of DE-miRNAs, further research was conducted on the upstream regulatory hsa-mir-449a of CCNB1.

### miR-449a inhibits SaOS-2 cell proliferation

**FIGURE 8:**
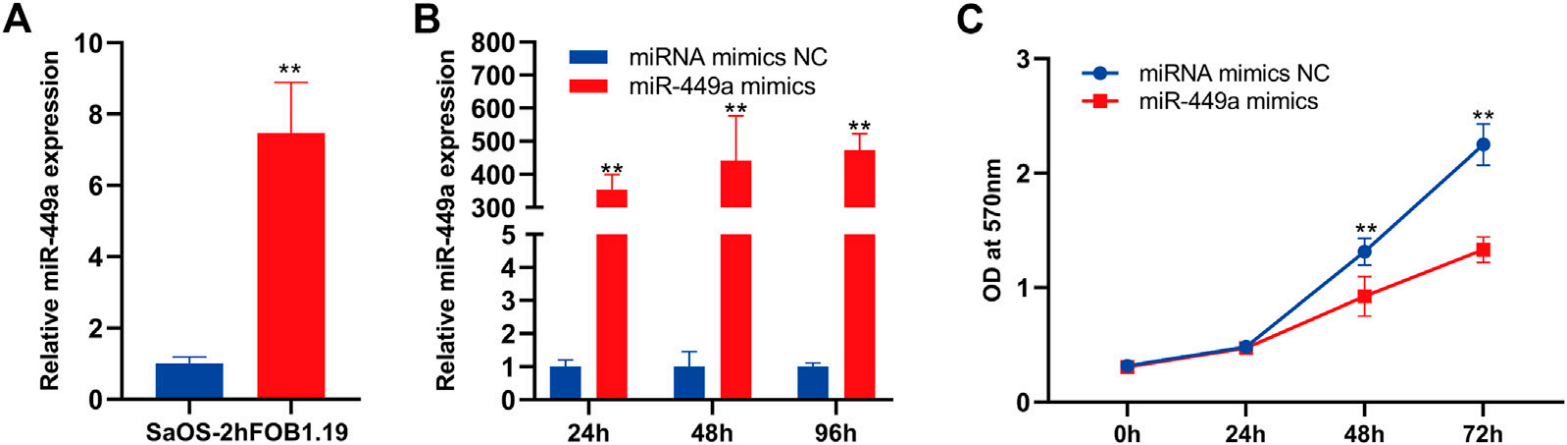
miR-449a inhibits proliferation of SaOS-2 cell. **(A)** RT-qPCR of the gene expression levels of miR-449a in hFOB 1.19 and SaOS-2 cells. **(B)** SaOS-2 cells were transfected with miR-449a mimics. **(C)** Following transfection, cell proliferation was analyzed by MTT assay.

The function of mir-449a in osteosarcoma was selected for further validation using wet experiments. First of all, Reverse transcription-quantitative PCR (RT-qPCR) was used to identificate mir-449a expression in hFOB 1.19 and SaOS-2 cells. In contrast to SaOS-2 cells, miR-449a are expressed at higher levels in hFOB 1.19 lines (Figure 8A). In the next step, we overexpressed miR-449a in SaOS-2 cells by transfection with mimics and evaluated gene expression after 24 h, 48 h, and 72 h. MiR-449a expression was significantly increased in SaOS-2 cells transfected with miR-449a mimics for 24, 48, and 72 compared with SaOS-2 cells transfected with respective NCs (Figure 8B). MTT assays were used to determine whether SaOS-2 overexpression affected SaOS-2 cells proliferation. The results of Figure 8C showed that miR-449a mimics significantly inhibited proliferation of osteosarcoma cells after 72 h.

### Inhibition of miR-449a promotes cell proliferation and inhibits apoptosis

**FIGURE 9:**
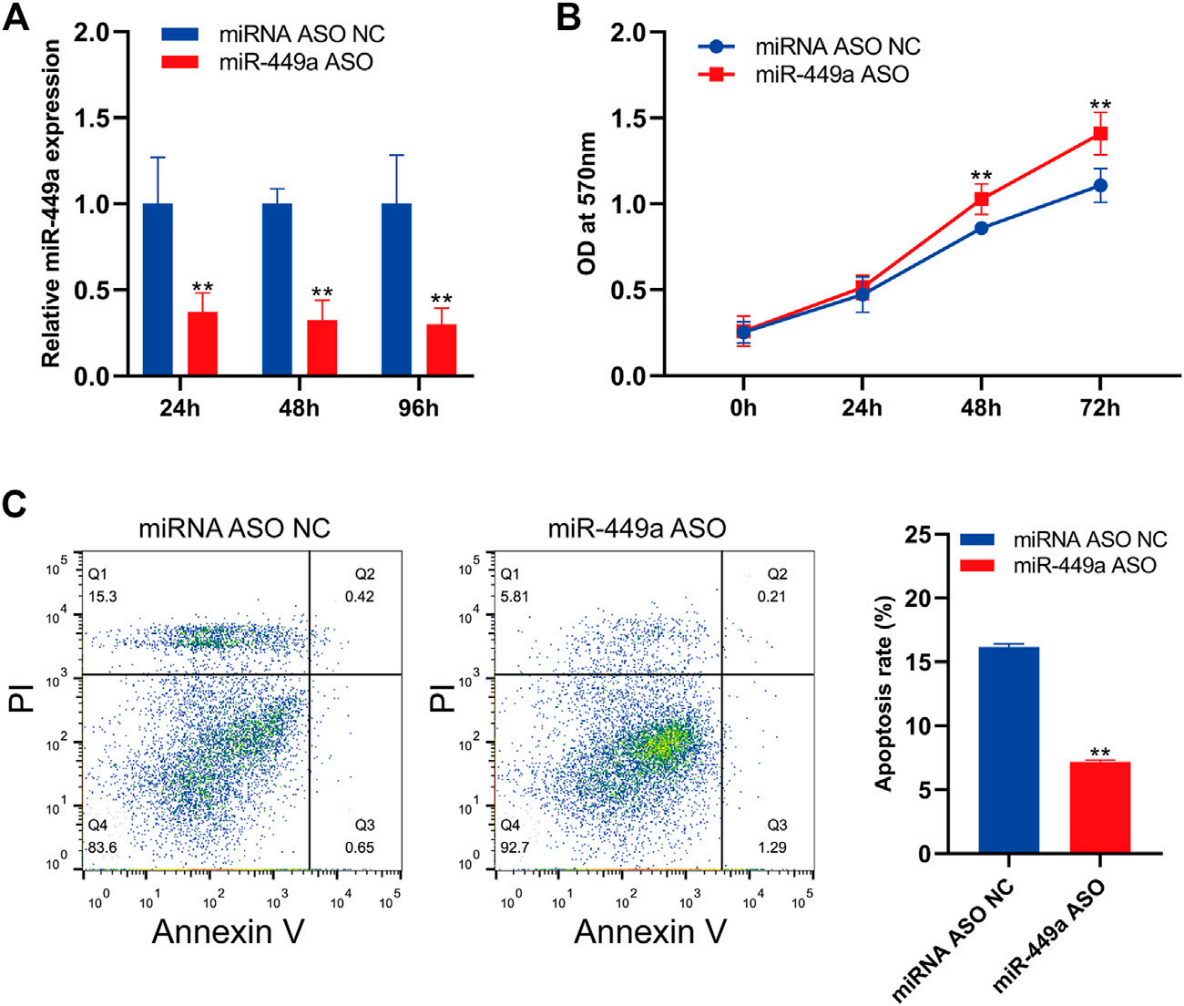
Inhibition of miR-449a promotes SaOS-2 cell proliferation and inhibits apoptosis. **(A)** SaOS-2 cells were transfected with miR-449a ASOs. **(B)** Following transfection, cell proliferation was analyzed by MTT assay. **(C)** Annexin V/PI double staining was performed 24 h after transfection with miR-449a ASO to analyze apoptosis rate.

The expression of mir-449a in SaOS-2 cells was inhibited by transfection with specific hsa-mir-449a ASO. By RT-qPCR, the expression of mir-449a in SaOS-2 cells was measured 24 h, 48 h, and 96 h after transfection. Hsa-miR-449a ASO transfection significantly reduced miR-449a expression at 24 h, 48 h, and 96 h (Figure 9A). Next, The cell proliferation of SaOS-2 cells was measured after 24 h, 48 h, and 72 h of miR-449a ASO transfection. Transfecting Saos-2 cells with hsa-mir-449a ASO promoted osteosarcoma cell proliferation (Figure 9B). After 24 h of hsa-mir-449a ASOs transfection, we performed an annexin V/PI double staining to determine cell apoptosis rates, and found that miR-449a ASOs transfection inhibited cell apoptosis rates (Figure 9C). These experiments showed that the Saos-2 OS cell line grew faster after miR-449a inhibition.

### miR-449a targets CCNB1

**FIGURE 10:**
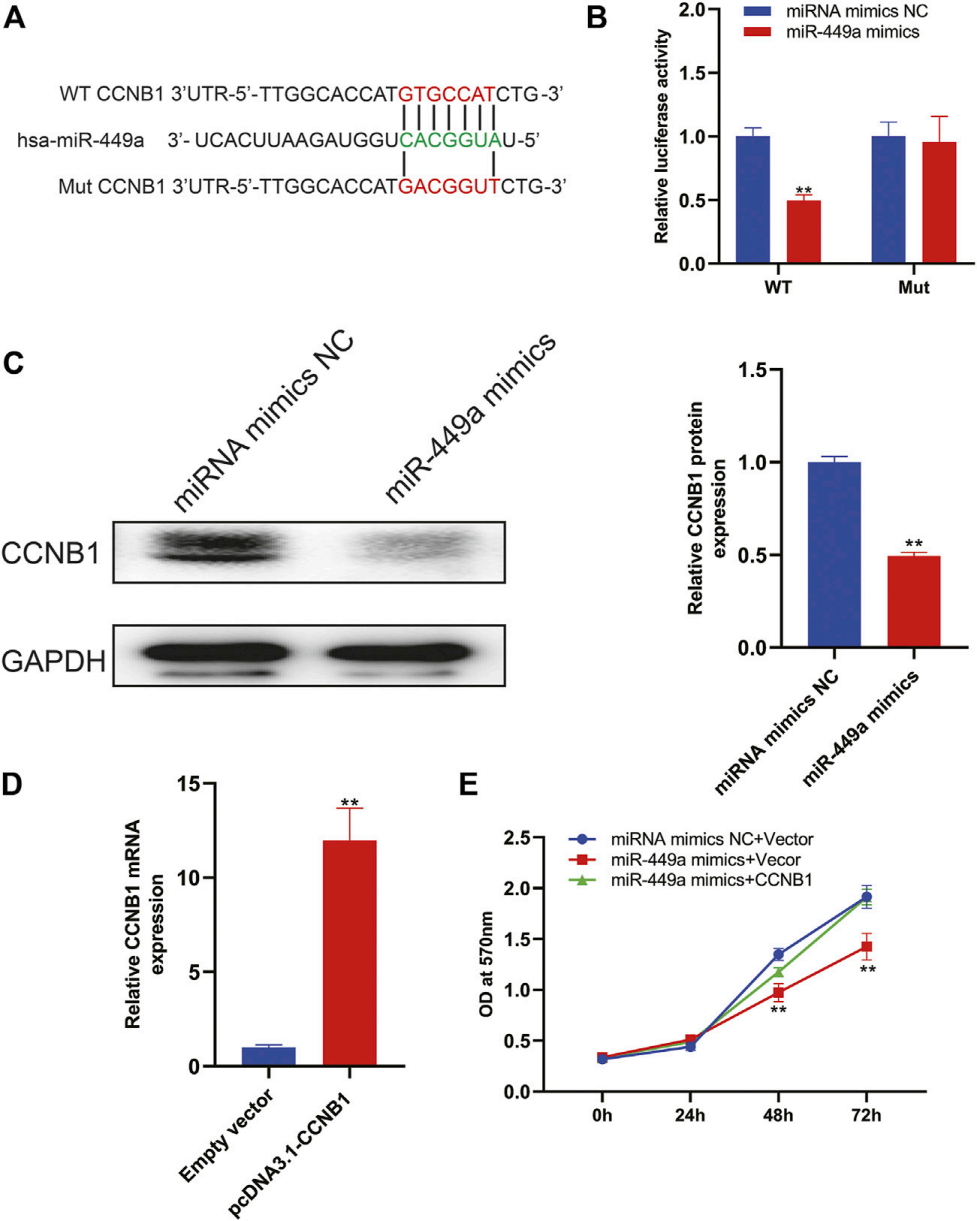
CCNB1 is downstream target of miR-449a. **(A)** MiR-449a binding sites and mutation sites. **(B)** MiR-449a mimics or miRNC and luciferase reporter vectors containing WT or mutant 3′UTRs were co-transfected in SaOS-2 cells. **(C)** Western blot analysis was performed after miR-449a mimics and miR-NC were transfected into cells. **(D)** Transfected SaOS2 cells with CCNB1 overexpression plasmid (pcDNA3.1-CCNB1) and RT-qPCR was performed to detect CCNB1 expression. **(E)** Following transfection, cell proliferation was analyzed by MTT assay.

Based on the above mentioned bioinformatic analysis, we reasoned that CCNB1 was a target of miR-449a. Using TargetScan, we predicted the binding sites, as shown in Figure 10A. In the following step, we cloned wildtype and mutant 3′UTRs of CCNB1 and inserted them into luciferase reporter plasmids. According to the results of luciferase activity assay in Figure 10B, miR-449a mimics bind to CCNB1 and luciferase signal decreases when there was no mutation in 3′UTR region of CCNB1 gene. In contrast, mutations in miR-449a binding sites in CCNB1’s 3′-UTR abolished the luciferase activity. The expression of CCNB1 protein was then assessed by Western blotting after transfection with miR-449a mimics. Figure 10C showed that CCNB1 protein levels were dramatically decreased after transfection with miR-449a mimics in SaOS-2 cells. Lastly, our experiments were conducted using SaOS-2 cells transfected with miR-449a mimic and CCNB1 overexpression plasmid (pcDNA3.1- CCNB1). RT-qPCR was used to confirm the effect of pcDNA3.1- CCNB1 (Figure 10D). Based on MTT assays of the CCNB1 overexpressing cells after miR-449a mimic transfection (Figure 10E), CCNB1 overexpression reversed miR-449a mimics’ proliferative effect.

## Discussion

Despite a lot of studies have been demonstrated that miRNA plays a significant role not only in the tumors development but also in tumors progression, associations between miRNAs and their mRNA targets are unknown or not well understood (Rupaimoole and Slack, 2017). Using bioinformatics tools, miRNA-mRNA regulatory networks were analyzed to understand the function and mechanism of miRNAs in OS.

On the basis of GSE65071 downloaded from GEO, 10 samples from primary OS and 15 samples from healthy control individuals were screened. 212 DE-miRNAs between OS and control samples were screened out, including 38 up-regulated and 174 down-regulated miRNAs.Then, two up-regulated miRNAs (hsa-mir-328, hsa-mir-107) and seven down-regulated miRNAs (hsa-mir-133b, hsa-mir-206, hsa-mir-1-3p, hsa-mir-133a, hsa-mir-449a, hsa-mir-181daysay, hsa-mir-134) were selected based on the results of comparison between DE-miRNAs and miRNet 2.0 Disease database. In general, our results are the same with those reported in the literature. MiR-1-3p could influence bone mass by regulating bone resorption, which was associated with osteosarcoma microenvironment (Gu et al., 2020). MiR-107 inhibited osteosarcoma cell proliferation *via* targeting SALL4 (Shi et al., 2022). MiR-133b was also linked with the pathogenesis of osteosarcoma (Mutlu et al., 2021). The exosomal miR-206 derived from bone marrow mesenchymal stem cells inhibits osteosarcoma progression by targeting TRA2B (Zhang et al., 2020). OS cell proliferation and invasion was inhibited by miR-133a by targeting IGF-1R (Gao et al., 2016). The miR-134–5p/KRAS Axis was strongly associated with the Development of osteosarcoma (Zhang et al., 2021).

Several studies have shown significant interactions between transcription factors and miRNA expression (Stavast et al., 2018; Han et al., 2022). Transcription factors and miRNA play a number of roles in regulating mRNA transcription and translation. We predicted up-stream TFs of DE-miRNAs using FunRich (e.g., SP1, SP4, KLF7, YY1, EGR1). Transcription Factor SP1 plays a role in many processes in the cell. Chou et al. reported that dihydromyricetin suppresses osteosarcoma *via* SP-1 signaling axis (Chou et al., 2021). The Kruppel-like factor 11 (KLF11) is expressed in a wide variety of tissues and controls proliferation, differentiation, and apoptosis (Wang et al., 2021b). MiR-3666/KLF7 axis, as the downstream target of KCNQ1OT1, regulate tumor growth (Huang et al., 2021). The zinc finger protein YY1 belongs to the GLI-Kruppel class and is widely distributed throughout the human body (Kwiatkowska et al., 2022). Early growth response protein-1 (EGR1) encodes a zinc finger protein of the EGR family, which is involved in transcriptional regulation (Wang et al., 2021c). Based on the findings of Han et al., LINC00857/miR-150–5p/c-Myc modulated anticancer effects of Scutellarin *via* EGR1 (Han et al., 2021).

By comparing target genes predicted from DE-miRNAs with DEGs, we identified 12 down-regulated and 310 up-regulated mRNAs. The GO functional annotation of 322 DEGs found that BP-GO terms were enriched in sister chromatid segregation, nuclear chromosome segregation, and chromosome segregation. Several studies have supported this fingding, including Wang et al. (Meyer et al., 2009). The presence of separate overexpression and aberrant nuclear localization is common in many tumor types including OS and may be a predictor of survival. For CC terms, DEGs were enriched in chromosomal region, fibrillar collagen trimer, and banded collagen fibril. Hoshi found that osteosarcoma cells produce collagen fibrils (Hoshi, 2014). MF terms of these genes were mostly enriched in extracellular matrix structural constituent, single-stranded DNA binding, and extracellular matrix structural constituent conferring tensile strength. This finding is in line with the characteristics of OS. Because osteosarcoma is characterized by its signature production of extracellular matrix (osteoid) by tumor cells, the MF results fits this characterization (Cui et al., 2020). DEGs are significantly enriched in nine KEGG pathways, and most of them were enriched in Cell cycle, Spliceosome, and Protein digestion and absorption. These pathways identified in osteosarcoma have previously been implicated, but novel genes may drive these pathways in human osteosarcoma and represent the potential OS pathogenesisand OS therapeutic targets (Zhang et al., 2019; Wang et al., 2020a; Liu et al., 2021; Li et al., 2022).

By using PPI network and topological analysis algorithm, hub genes (CCNB1 and CDK1) were identified. We further used GEPIA to screened CCNB1 as key genes in OS. CCNB1’s upstream regulatory hsa-mir-449a was then studied. We found that miR-449a inhibited proliferation of Saos-2 cells in our study. Additionally, miR-449a inhibition decreased osteosarcoma cell apoptosis. The conclusion from silico analysis was further validated by luciferase reporter assay. CCNB1 had been shown to be a downstream target of miR-449a. To the best of our knowledge, this was the first Chinese research to combine wet and dry laboratory experiments to study miR-449a target gene CCNB1 in osteosarcoma.

The miR-449 family consists of three members (miR-449a, miR-449b, and miR-449c) encoded by a cluster located on chromosome 5q11.2 in CDC20B, which has been associated with cancer susceptibility (Yong-Ming et al., 2017). The evidence for this has been demonstrated in many studies. However different cancer types have similar roles for miR-449a in cancer progression (Wang et al., 2018). Overexpression of miR-449a significantly suppress the progression of colorectal cancer by affecting the expression of target genes LEF-1 and cyclin D1 (Lan et al., 2021). At the same time, SATB1 is a target of miR-449a that inhibits HCC invasion and promotes apoptosis (Li et al., 2020). Taken together, miR-449a may be useful for the treatment of cancer, especially osteosarcoma.

To better explore the function of miR-449, we investigated its downstream target genes CCNB1. CCNB1 is an essential protein for cell proliferation, playing a role in mitosis (Zhang et al., 2018). Wang et al. found that miR-718 inhibit lung cancer by targeting CCNB1 (Wang et al., 2020b). According to other study, miR-144/CCNB1 was a critical factor in HCC (Gu et al., 2019). Yu et al. had proven that CircCCNB1 sponged miR-449a to inhibit cellular senescence by targeting CCNE2 (Yu et al., 2019). So we thought CCNB1 is one of the important miRNA-target in tumor. It was in line with our findings in OS. But there was still no clear understanding of the mechanism by which miR-449a /CCNB1 worked in osteosarcoma. Based *in silico* predictions, the mechanism might be related to the Cell cycle, Spliceosome, and Protein digestion and absorption in osteosarcoma. The conclusion of this study was in accordance with that of other studies in the literature. CCNB1 mechanism in HCC influenced Cell cycle through PI3K and AKT phosphorylation (Xia et al., 2021).

It is important to note, however, that this study has some limitations. First is the selection of cell line. SaOS-2 and hFOB 1.19 cells lack both intact p53 and Rb (Li et al., 2021b). It is also important to note that no *in vivo* studies were performed in this study. As a final point, further research is needed to determine the cellular mechanism responsible for miR-449a /CCNB1 axis.

This article investigated the effect of miR-449a /CCNB1 axis on osteosarcoma through using bioinformatics analysis and “wet” experiments. The analysis has important clinical implications for the selection of osteosarcoma therapeutic targets. MiR-449a could be used as anticancer agents loaded in engineered exosomes to suppresses osteosarcoma. There have been some studies that the engineered exosomes as vehicles for the delivery of miRNA into tumor as a new treatment for cancer (Liang et al., 2020; Wang et al., 2021d; Zhou et al., 2021). As a whole, we believed that miR-449a was used for engineered exosome-based treatments to suppresses OS cell proliferation by targeting CCNB1.

## Data Availability

The datasets presented in this study can be found in online repositories. The names of the repository/repositories and accession number(s) can be found in the article/supplementary material.
