# Corrigendum: Screening for MicroRNA combination with engineered exosomes as a new tool against osteosarcoma in elderly patients

**DOI:** 10.3389/fbioe.2025.1615190

**Published:** 2025-05-06

**Authors:** Jiyu Han, Zitong Zhao, Yanhong Wang, Tao Yu, Daqian Wan

**Affiliations:** ^1^ Department of Orthopedics, Tongji Hospital, School of Medicine, Tongji University, Shanghai, China; ^2^ Key Laboratory of Spine and Spinal Cord Injury Repair and Regeneration, Ministry of Education, Shanghai, China; ^3^ Department of Orthopaedic, Ruijin Hospital, Shanghai Jiao Tong University School of Medicine, Shanghai, China

**Keywords:** osteosarcoma, miR-449a, CCNB1, engineered exosomes, bioinformatics analysis, wet-lab experiment

In the published article, there was an error in affiliation [1]. Instead of “School of Medicine, Department of Orthopedics, Tongji Hospital, Tongji University, Shanghai, China”, it should be “Department of Orthopedics, Tongji Hospital, School of Medicine, Tongji University, Shanghai, China”.

In the published article, there was an error in the author list. The symbol † was erroneously included for authors Tao Yu and Daqian Wan. The corrected author list appears below.

“Jiyu Han^1,2,†^, Zitong Zhao^2,†^, Yanhong Wang^1,2,†^, Tao Yu^3,^* and Daqian Wan^1,2,^ *”

The authors apologize for this error and state that this does not change the scientific conclusions of the article in any way. The original article has been updated.

