# Supplementary material for: Screening for MicroRNA combination with engineered exosomes as a new tool against osteosarcoma in elderly patients
Source: Front Bioeng Biotechnol. 2022 Dec 5;10:1052252. doi: 10.3389/fbioe.2022.1052252 (PMC9760984; doi:10.3389/fbioe.2022.1052252)
Supplement: Supplementary file 1 [file DataSheet1.docx]

https://www.jianguoyun.com/p/DcVsvS0QyJH8Chio3NoEIAA
